# Query on Bleeding in Retention of Urine

**Published:** 1813-04

**Authors:** George Hume Weatherhead

**Affiliations:** Surgeon, R. N.


### *To the Editors of the Medical and Physical Journal.*

GENTLEMEN,

BY inserting the following Query in your valuable Journal, for the consideration of those whose means of ascertaining the propriety of its being applied practically are more extensive than my own, will infinitely oblige

Your very humble Servant,
GEORGE HUME WEATHERHEAD,
Surgeon, R. N.

QUERY.—Whether, in cases of retention of urine from inflammation of the neck of the bladder, wherein the usual means have been employed without success, and the operation is deemed the only resource; might not venesection *ad deliquium* enable the surgeon, during the general relaxation that attends the state of syncope, to introduce a flexible gum catheter, and relieve the bladder by the evacuation of the urine?

What has induced me to put this query, was the happy result of a case thus treated, in which I succeeded in discharging the urine, and relieved my patient of every dangerous symptom. I left the catheter in the bladder without occasioning any intolerable irritation, and the antiphlogistic plan was of course adopted.

Nay, it may be asked if this method to empty the bladder might not be pursued at a much earlier period of the disease; for, from whatever cause the inflammation may originate, there can be no doubt that, as the urine accumulates, and as the bladder distends, our prospect of subduing the inflammation, and thereby discharging the urine, becomes less promising and more difficult.

The irritation excited by the presence of an elastic gum catheter, in a natural canal, must assuredly be less than that consequent on a puncture through the substance of the bladder, and the presence of a silver canula in a preternatural passage.

In trying the experiment, the patient ought to be bled in an upright posture, and from a large orifice, the catheter having been previously oiled, and every thing arranged for its convenient introduction.

12, *Broad-street, Soho,*

*Feb.* 16, 1813.

